# miR-205 inhibits cell growth by targeting AKT-mTOR signaling in progesterone-resistant endometrial cancer Ishikawa cells

**DOI:** 10.18632/oncotarget.15886

**Published:** 2017-03-03

**Authors:** Zhihong Zhuo, Huimin Yu

**Affiliations:** ^1^ Ningbo No. 2 Hospital, 315010 Ningbo, People's Republic of China

**Keywords:** microRNA, endometrial carcinoma, autophagy, progesterone resistance

## Abstract

**Purpose:**

miR-205 is significantly up-regulated in endometrioid adenocarcinoma. In this study, the significant anticancer effect of a miR-205 inhibitor was investigated in both endometrial carcinoma and progesterone-resistant endometrial carcinoma cells.

**Results:**

Compared with Ishikawa endometrial cancer cells, miR-205 was expressed at higher levels in a progesterone-resistant (PR) sub-cell line. Inhibition of miR-205 suppressed the growth of cancer cells in a dose- and time-dependent manner. Moreover, the miR-205 inhibitor induced a marked increase in the percentage of Ishikawa-PR cells in G2/M phases and a decrease in the percentage of cells in G0/G1 and S phases. In addition, miR-205 inhibitor-treated tumor cells exhibited increased apoptosis. Moreover, miR-205 was found to negatively regulate PTEN expression and lead to autophagy and activation of the AKT/mTOR pathway in PR cells, and PTEN protein levels significantly decreased with development of progesterone resistance in endometrial cancer cells. Western blot assay showed up-regulated autophagy, as indicated by expression of LC3-II/LC3-I and beclin1, in Ishikawa cells; in particular, autophagy was markedly induced in PR cells treated with the miR-205 inhibitor.

**Materials and Methods:**

We measured and analyzed cell growth curves with and without miR-205 inhibition with the MTT assay, miR-205 expression by qRT-PCR, cell cycle and apoptosis using annexin V/propidium iodide staining and flow cytometry, and autophagy, apoptosis, and AKT-mTOR signaling by western blotting.

**Conclusions:**

Inhibition of miR-205, which targets the AKT-mTOR pathway, in endometrial cancer cells provides a potential, new treatment for PR endometrial carcinoma.

## INTRODUCTION

The incidence of endometrial cancer (EC), one of the most common genital malignancies in women, is increasing rapidly worldwide [1, 2]. The prognosis of EC, even in the early stage, is poor, yet the factors contributing to the risk of EC are not fully understood. A high level of endogenous estrogen is one of the primary causes of EC, and progesterone can be used to inhibit and reverse the cancer-promoting effects of estrogen. At present, the main conservative treatment for atypical endometrial hyperplasia and endometrial adenocarcinoma is a long-term, large-dose regimen of progesterone, an approach that is strictly limited to patients who have reliable follow-up conditions, no drug contraindications with hormones and anticancer application, or are young patients with a strong desire to remain fertile [3, 4]. As the pathogenesis of EC with progesterone resistance is unclear clinically, there is an urgent need to find new therapeutic targets and strategies for treating EC, particularly in these patients. The therapeutic effect of progesterone treatment has prompted investigations into the requirement of a large dose and exploration of the mechanism; in addition, study of cellular resistance to progesterone is of great significance for clinical treatment of EC [5]. Nonetheless, the mechanism by which long-term application of progesterone inhibits EC cell proliferation, invasion and metastasis remains poorly understood.

MicroRNAs are endogenous, non-coding small RNAs comprising a single strand of 18–25 nucleotides. Complete pairing causes the degradation of a target mRNA, whereas incomplete pairing inhibits translation and thus alters gene expression posttranscriptionally [6, 7]. Various tissues and cells express different miRNA spectra at different stages of development [8]. Although the miRNA gene itself is not translated into protein, by regulating the expression of other genes, miRNAs exert an effect on cellular activity, especially the cell cycle in terms of growth, proliferation, differentiation and apoptosis. Indeed, miRNAs have recently become a hot issue in oncology because they are related to the occurrence and development of tumors. For example, abnormal expression of miRNAs is important for the development of tumors [9], with roles in EC [10–13], and miRNAs can act as either oncogenes or tumor suppressor genes in tumourigenesis. miR-205 dysregulation occurs in EC tissue [14]. Chung and colleagues [15] showed that that miR-205 expression in endometrial carcinoma was 27.2 times that in normal endometrium and that antisense inhibition decreased miR-205 expression by 64.9%. Wu [16] found that expression of miR-205 in endometrioid adenocarcinoma and adjacent tissues was increased by 18.9-fold, and another study reported similar results [17, 18], showing that miR-205 expression was higher in EC. In addition, it has been reported that expression of miR-205 in EC is significantly higher than that in normal endometrium [19]. These results suggest that high expression of miRNA-205 is associated with the occurrence of EC.

We hypothesized that expression of miRNA-205 in progesterone-resistant (PR) EC is increased and related to the function of the targeted gene. More specifically, the exact mechanism by which miR-205 induces autophagy in EC, especially PR-EC, remains to be elucidated. The objective of this study was to demonstrate that inhibition of miR-205 would suppress proliferation of PR-EC Ishikawa cells. Here, we show that miR-205 inhibition induces autophagy and targets AKT-mTOR signaling in the apoptosis pathway. We sought to discover the effects of miR-205 on EC cell viability and to investigate the molecular mechanisms by which miR-205 exerts its action on PR-EC cells.

## RESULTS

### miR-205 is expressed at higher levels in Ishikawa-PR cells compared with Ishikawa cells

To examine whether miR-205 is differentially expressed between Ishikawa and Ishikawa-PR cell lines, we measured miR-205 expression levels by qRT-PCR. As shown in Table 1, Ishikawa-PR cells exhibited high miR-205 expression, whereas low miR-205 expression was detected in Ishikawa cells. The difference was statistically significant (*p* < 0.05).

**Table 1:** The expression of miR-205 between Ishikawa-PR cells and Ishikawa cells

		Ishikawa cells	Ishikawa-PR cells
U6	average Ct(mean, SD)	15.168,0.196	15.462,0.060
	average relative copies	1.40E + 09	1.15E + 09
miR-205	average Ct(mean, SD)	21.651,0.176	21.287,0.096
	average relative copies	1.77E + 07	2.26E + 07
⊿Ct		6.482	5.825
2^(−⊿Ct)^		0.011	0.018
relative ratio		0.013	0.020

qRT-PCR verified that miR-205 was highly expressed in Ishikawa-PR cells compared with Ishikawa cells (p < 0.05). The results are presented as the means ± SD of three independent experiments.

⊿Ct = Ct value of U6- Ct value of miR-205.

relative ratio = average relative copies of U6/average relative copies of miR-205.

To further assess endogenous levels of miR-205, qRT-PCR analysis was used to detect miR-205 expression with or without miR-205 inhibitor treatment in Ishikawa cells and Ishikawa-PR cells. As expected, miR-205 mRNA levels were significantly decreased in cells treated with the miR-205 inhibitor (Table 2); in particular, the value of 2^(−⊿Ct)^ increased from 0.011 and 0.018 to 0.004 and 0.008 in Ishikawa cells and Ishikawa-PR cells, respectively. More importantly, the relative ratios increased from 0.013 and 0.020 to 0.004 and 0.009 in both cell lines. These data indicated that miR-205 expression is inversely deregulated by miR-205 in EC cells and that miR-205 in involved in progesterone resistance in EC.

**Table 2:** The expression of miR-205 with miR-205 inhibitor between Ishikawa-PR cells and Ishikawa cells

		Ishikawa cells	Ishikawa-PR cells
U6	average Ct(mean, SD)	14.471,0.206	15.423,0.163
	average relative copies	2.23E + 09	1.18E + 09
miR-205	average Ct(mean, SD)	22.616,0.089	22.432,0.114
	average relative copies	9.24E + 06	1.05E + 07
⊿Ct		8.146	7.008
2^(−⊿Ct)^		0.004	0.008
relative ratio		0.004	0.009

The results are presented as the means ± SD of three independent experiments.

⊿Ct = Ct value of U6- Ct value of miR-205.

relative ratio = average relative copies of U6/average relative copies of miR-205.

### The miR-205 inhibitor inhibits miR-205 expression in Ishikawa-PR cells in a time- and dose-dependent manner

**Figure 1:**
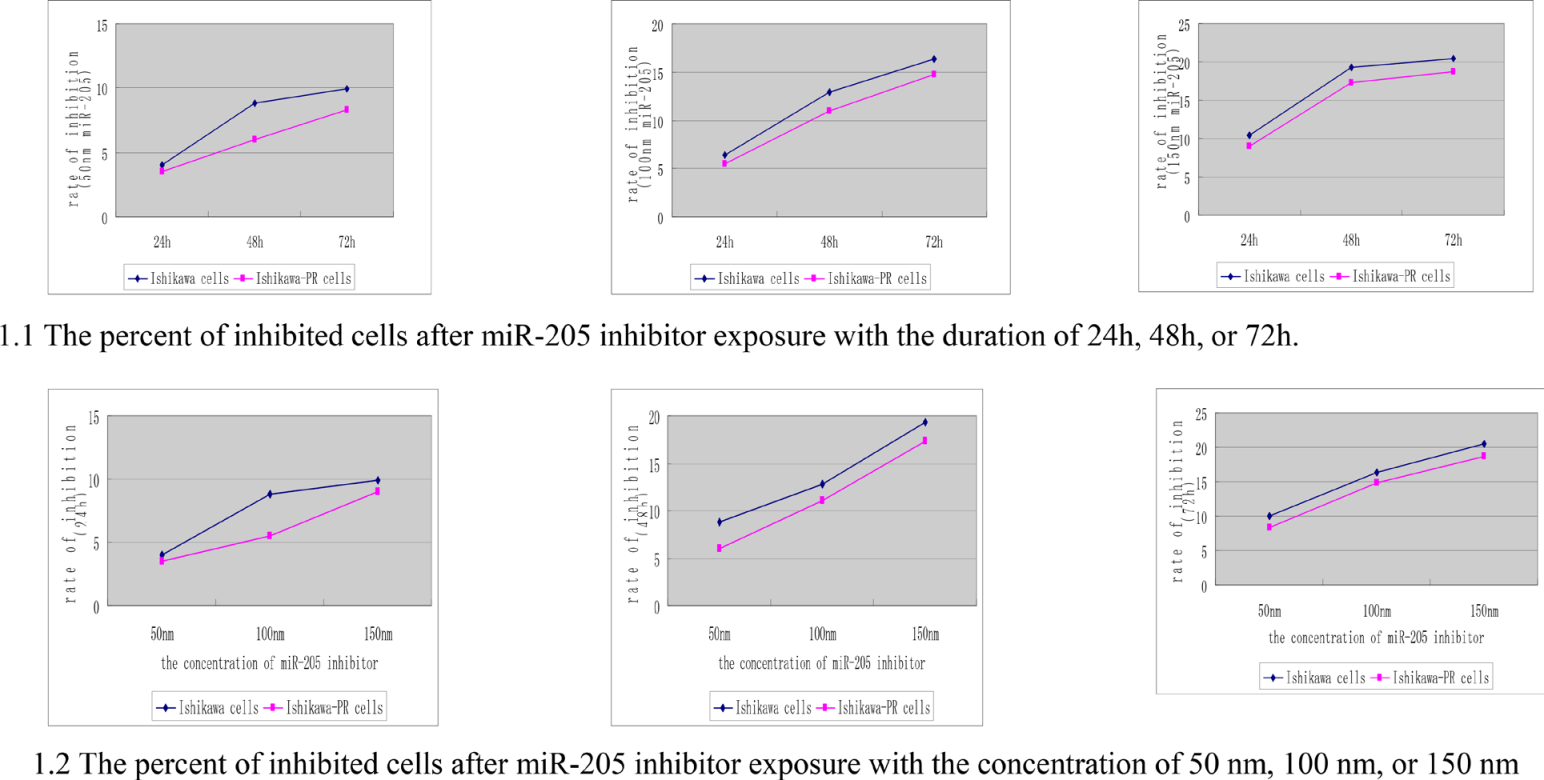
The cell growth inhibition of the Ishikawa cells and Ishikawa-PR cells with a time- and dose-increase manner

We used the MTT assay to determine whether the miR-205 inhibitor affects cell growth of Ishikawa and the Ishikawa-PR cells differently. We first evaluated the effect of different concentrations of miR-205 inhibitor and different durations (24, 48 and 72 h) on cell growth and then plotted the data obtained using SPSS software (Figure 1). Regardless of whether the concentration was 50 nM, 100 nM, or 150 nM, the percentage of cells inhibited by the miR-205 inhibitor increased in a time-dependent manner. The results shown in Figure 1 indicate that the rate of cell growth suppression from 24 to 72 h was significantly enhanced in a dose-dependent manner (*p* < 0.05). Thus, we used 150 nM inhibitor for all ensuing experiments.

### miR-205 inhibitor arrests the cell cycle at G2/M phase and induces apoptosis in Ishikawa-PR cells

**Figure 2:**
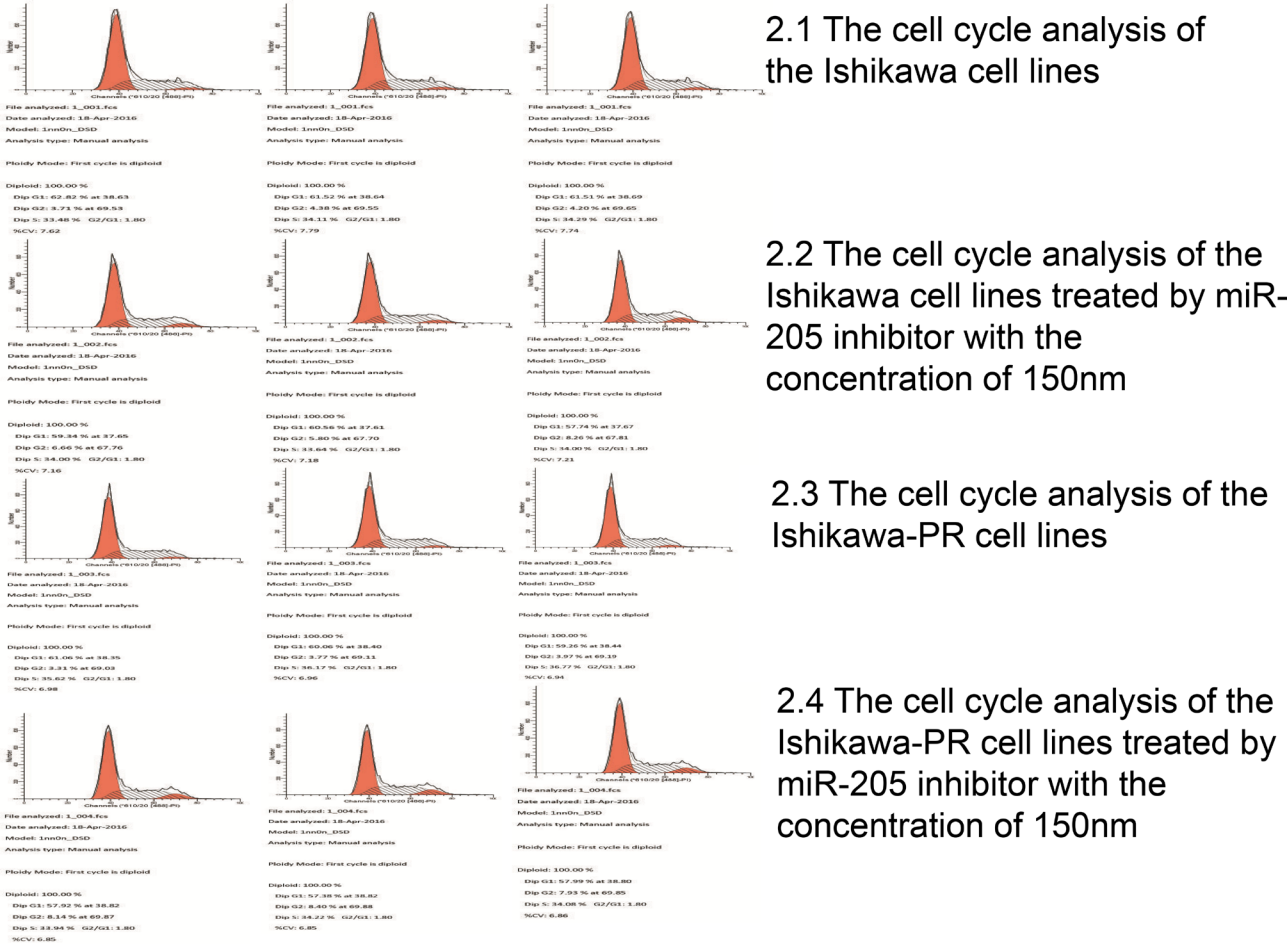
The cell cycle of the Ishikawa cells and Ishikawa-PR cells using propidium iodide binding assay by FACS

Given that miR-205 may have an oncogenic effects on EC, we considered whether miR-205 might have an important function in cell cycle arrest or apoptosis in EC cells. We verified that the growth inhibition observed in both cell lines treated with the inhibitor was due to changes in the cell cycle. Ishikawa and Ishikawa-PR cells were incubated with 150 nM inhibitor for 48 h, and cell cycle profiles at G0/G1, G2/M and S phases were measured by PI staining and flow cytometric analysis (Figure 2). We observed an increase in the percentage of cells in S phase (*p* = 0.01) but no significantly different changes in the percentage of cells in G0/G1 and G2/M phases (*p* = 0 .06, *p* = 0.21) between the Ishikawa cells and Ishikawa-PR cells. Most importantly, the inhibitor induced Ishikawa cells to arrest in G2/M phase (*p* = 0.02) and a marked increase in the percentage of Ishikawa-PR cells in G2/M phase but a decrease in the percentage of Ishikawa-PR cells in G0/G1and S phases (Table 3, *p* < 0.05).

**Table 3:** Cell-cycle analysis measured by propidium iodide staining and flow cytometric analysis of stained cells was performed with a FACScan

	Ishikawa cells			Ishikawa-PR cells		
miR-205 inhibitor	G0/G1(mean, S)	G2/M(mean, S)	S(mean, S)	G0/G1(mean, S)	G2/M(mean, S)	S(mean, S)
0 nM	61.95,0.75	4.10,0.35	33.96,0.43	60.12,0.90	3.68,0.34	36.19,0.58
150 nM	59.21,1.41	6.91,1.25	33.88,0.21	57.76,0.33	8.16,0.24	34.08,0.14
p	0.05	0.02	0.78	0.01	0.00	0.00

**Figure 3:**
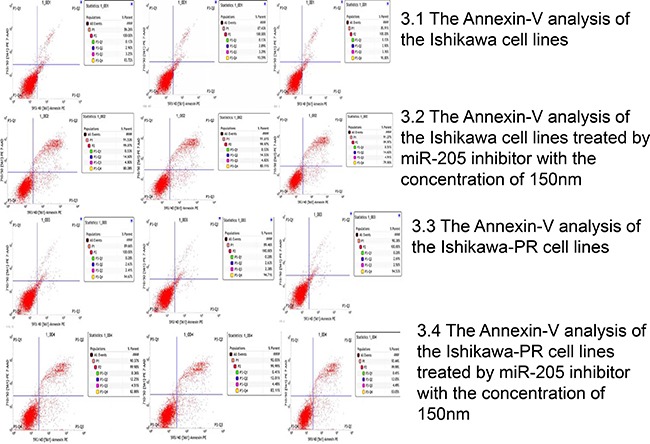
The cell apoptosis of the Ishikawa cells and Ishikawa-PR cells using an annexin-V and propidium iodide binding assay by FACS

We then evaluated whether inhibition of cell growth and viability was due to stimulation of apoptosis using an annexin-V and PI assay; the results are shown in Figure 3. We found a significant induction of early and late apoptosis or the death phase between the two cell lines (*p* < 0.05). We detected a significant increase in the annexin-V/propidium iodide (+/−)-stained subpopulation after 48 h of treatment with 150 nM inhibitor in both cell lines (3.27 ± 0.12% versus 4.84 ± 0.59%, 2.43 ± 0.06% versus 4.49 ± 0.15%, respectively). Moreover, the annexin V/propidium iodide (+/+)-stained fraction of Ishikawa and Ishikawa-PR cells was 2.90 ± 0.06% and 2.65 ± 0.03% and increased to 14.59 ± 0.05% and 12.10 ± 0.13%, respectively, after 48 h of incubation with the inhibitor (Table 4).

**Table 4:** Cell apoptosis analysis was measured by Annexin V and propidium iodide staining with flow cytometric analysis performed

	Ishikawa cells (mean, S)(Annexin V/propidium)				Ishikawa-PR cells (mean, S)(Annexin V/propidium)			
miR-205 inhibitor	−/+	+/+	+/−	−/−	−/+	+/+	+/−	−/−
0 nM	0.13, 0.00	2.90, 0.06	3.27, 0.12	93.70, 0.11	0.28, 0.00	2.65, 0.03	2.43, 0.06	94.64, 0.09
150 nM	0.52, 0.12	14.59, 0.05	4.84, 0.59	80.05, 0.09	0.39, 0.03	12.10, 0.13	4.49, 0.15	83.01, 0.12
p	0.00	0.00	0.00	0.00	0.00	0.00	0.00	0.00

### miR-205 negatively regulates PTEN expression during the development of progesterone resistance and leads to autophagy with activation of the AKT/mTOR pathway

To identify the relationship between miR-205 and PTEN levels in progesterone resistance, we employed western blotting to detect miR-205 and PTEN expression levels in Ishikawa and Ishikawa-PR cells. PTEN protein levels were significantly decreased in PR-EC cells, and treatment with the miR-205 inhibitor increased PTEN expression, especially in Ishikawa-PR cells.

**Figure 4:**
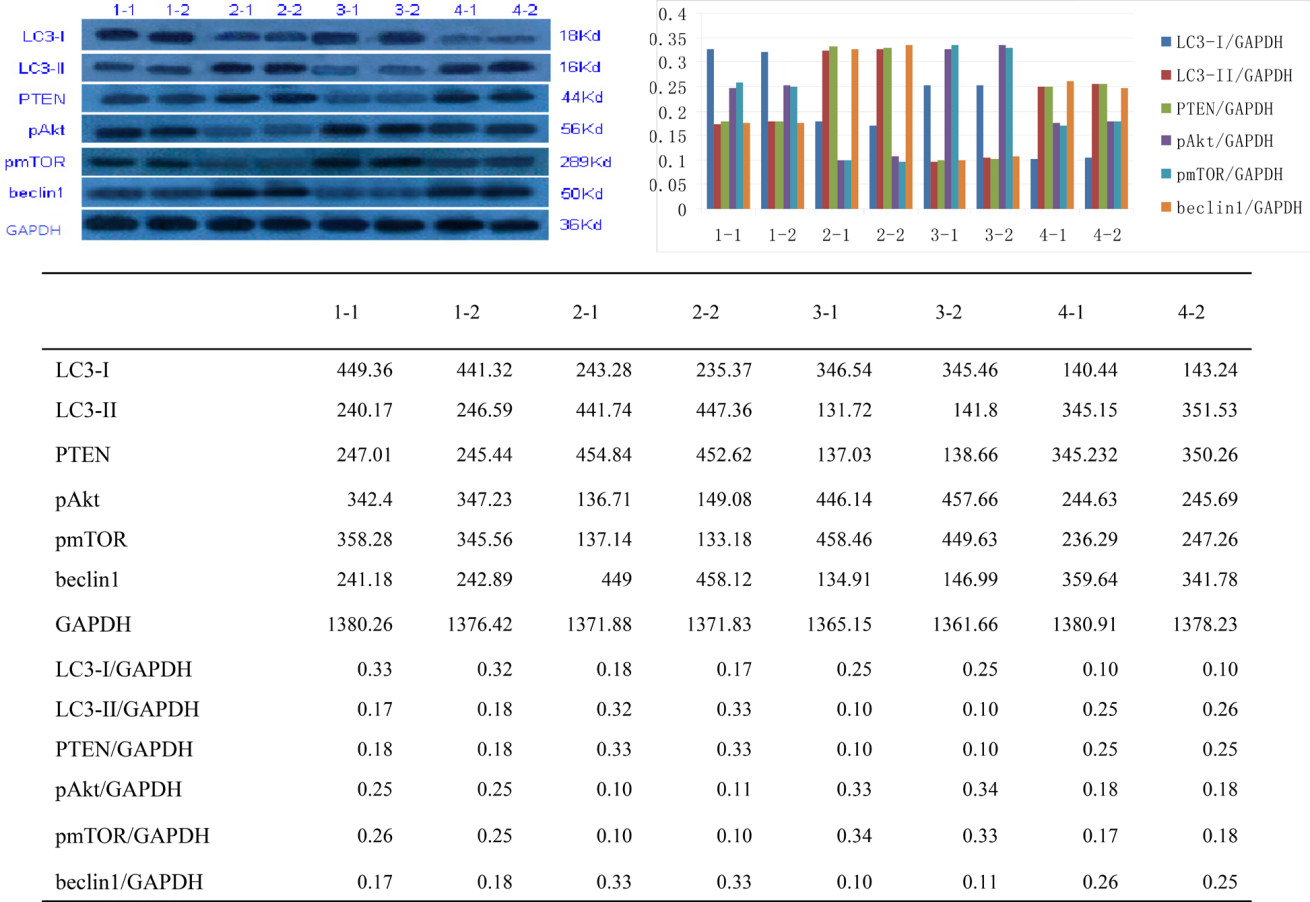
The expression of LC3-I, LC3-II, PTEN, p-AKT, pmTOR and beclin1 1-1,1-2: Ishikawa cells; 2-1, 2-2: Ishikawa-PR cells; 3-1, 3-2: Ishikawa cells with miR-205 inhibitor; 4-1, 4-2: Ishikawa-PR cells with miR-205 inhibitor

To further examine effects on the AKT/mTOR pathway and several downstream targets of miR-205, we measured expression of phosphorylated AKT (pAKT) and phosphorylated mTOR (pmTOR), the critical downstream targets of PTEN, and found them to be dramatically decreased with miR-205 inhibition (Figure 4). In addition, the expression levels of LC3-II/LC3-I and beclin1, potential functional downstream targets during autophagy, were increased by reduced expression of miR-205, particularly in Ishikawa-PR cells. Furthermore, our results indicated miR-205 inhibition to be inversely correlated with PTEN expression, leading to activation of the AKT/mTOR pathway in PR-EC cells. Moreover, inhibition of miR-205 expression markedly induced autophagy, as evidenced by up-regulation of LC3-II/LC3-I and beclin1, in Ishikawa cells, especially PR-EC cells.

## DISCUSSION

The discovery of microRNAs, novel gene regulatory factors, has provided a new area of study with regard to gene regulation and disease development [20]. Increased expression of miR-205 is consistently observed in EC compared with normal tissues [21], though it remains unknown whether inhibition of miR-205 can reverse progesterone resistance in EC. In the present study, we confirmed the anti-cancer function of a miR-205 inhibitor in Ishikawa cells, especially in progesterone resistance.

We observed miR-205 to be expressed at higher levels in Ishikawa-PR cells compared with Ishikawa cells. Furthermore, treatment with a miR-205 inhibitor markedly suppressed the growth of Ishikawa cells and PR cancer cells. Thus, miR-205 might be involved in progesterone resistance in EC, though the mechanism of progesterone resistance in EC remains unclear. In particular, it is unknown how apoptosis, estrogen and progesterone receptor and other pathways, which promote estrogen receptor (ER) and progesterone receptor (PR) expression and changes in tumor suppressor gene expression, increase expression of endogenous growth factors and receptors as well as growth factor and extracellular matrix secretion by stromal cells, leading to the occurrence of hormone-resistant EC [22, 23].

Moreover, this is the first study to demonstrate how miR-205 induction of cell growth is linked to apoptosis, cell cycle arrest, and autophagy processes in PR-EC cells. Using the MTT assay, we provide evidence for the dose- and time-dependent anti-tumor activity of a miR-205 inhibitor in EC cells and PR-EC cells. Apoptosis, cycle arrest and autophagy participate in pharmacologic inhibition. Because the tumorigenic activity of miR-205 is not completely clear, we investigated the effects of apoptosis and cycle arrest on the suppression of cell growth and proliferation. The miR-205 inhibitor induced Ishikawa cell cycle arrest in G2/M phase and increased the percentage of PR cells in G2/M phase but decreased the percentage of cells in G0/G1and S phases. Thus, the cancer-promoting effect of miR-205 in PR cells might occur through changes in the G2/M phase and G0/G1and S phases of the cell cycle. Accordingly, cycle-dependent kinase and its critical roles in cell cycle arrest and miR-205 activation in both cell lines should be studied. Annexin V is a phospholipid- and calcium-binding protein, and using the annexin-V staining method, we demonstrated the effectiveness of the miR-205 inhibitor (150 nM) in inducing early apoptotic, late apoptotic or necrotic events after 48 h of incubation in both cell lines. We observed elevations in the apoptotic phase in cancer cells treated with the inhibitor, with no marked changes even though the difference was significant. Our results show that a 48 h incubation with miR-205 inhibitor induced apoptosis in the PR-Ishikawa cell line. Nevertheless, Zhang et al. found that the rates of apoptosis in Ishikawa cells transfected with miR-205 mimics were significantly decreased compared with those of negative control miRNA-transfected cells [24]. This difference may be explained by differences in the experimental methods, concentrations, or duration of inhibitor incubation.

The PTEN gene is a selective tumor suppressor gene involved in cell adhesion, proliferation, and gene mutation in cancer tissues [25]. Samy et al. [26] found PTEN deletion in malignant gliomas, EC, melanomas, breast cancer and other tumors, indicating that the PTEN gene has an important function in suppressing transformation to a malignant tumor. The most commonly modified gene in EC is PTEN, which is located on human chromosome 10 and encodes a tyrosine kinase [27]. The simultaneous losses of PTEN lipid kinase and protein kinase activity lead to abnormal cell growth and escape from apoptosis, proliferation, and metastasis [28]. Approximately 83% of ECs and precancerous lesions exhibit decreased PTEN expression. Therefore, deletion of PTEN function is considered an early event in the development of endometrial tumors. Most PTEN mutations are heterozygous, without aleterd or lost protein function [29]. In high grades of cancer, 20% of PTEN mutations result in promoter methylation [30]. Therefore, PTEN-induced suppression of EC in early stages generally has a good prognosis, with a 5-year survival rate of approximately 80%. Overall, PTEN inactivation without mutation in EC has a 5-year survival rate of 50% [31]. In addition, our data reveal a new perspective regarding the regulatory mechanism of miR-205 on the PTEN gene in PR-EC. We showed that PTEN levels were significantly decreased in PR Ishikawa cells and that the miR-205 inhibitor increased PTEN expression in these cells. These findings suggest that miR-205 plays an important role in the loss of PTEN expression and the development of PR in EC cells.

PTEN has an important inhibitory function, promoting apoptosis and proliferation, and its deletion or mutation leads to tumor occurrence. We demonstrated that a miR-205 inhibitor caused enhanced p-AKT and pmTOR levels, the conversion of LC3-II/I, and beclin1 expression. We found that PTEN directly inhibited phosphatidylinositol 3, 4, 5-tris-phosphate (PIP3) or PI3K to negatively regulate Akt, thereby inhibiting mTOR in cell proliferation and apoptosis in the metabolic pathway [32]. Our results showed that the p-AKT and pmTOR protein levels were similarly enhanced. Both PI3K and mTOR can negatively regulate autophagy, thus further illuminating the mechanism by which PTEN inhibits the PI3K/Akt/mTOR pathway and promotes autophagy [33].

In autophagy, which was initially thought to be induced by a lack of nutrients in cells, the cytoplasmic components are transformed from organelles into energy for survival through digestion [34]. Autophagy is a multistep process that is subjected to strict regulation, and defects in autophagy result in tumors, muscle atrophy, neurodegenerative diseases and multiple skin diseases [35]. Autophagy is also involved in the immune response [36]. mTOR kinase is a key regulator of eukaryotic autophagy initiation, and the regulation of autophagy has gradually become the focus of tumor prevention and treatment research. mTOR is a serine/threonine kinase and a member of the PI3K kinase family that is involved in gene transcription, protein translation initiation, ribosome biogenesis, cell cycle and apoptosis and plays an important role in cell growth and differentiation [37]. To show that autophagy and miR-205 are connected in endometrial cells, especially PR-EC cells, we examined markers of autophagy and found increased conversion of LC3-I to LC3-II and beclin1 protein levels, especially in Ishikawa-PR cells. This suggests that Ishikawa-PR cells are even more sensitive to miR-205 inhibitor treatment than Ishikawa cells. In our next study, we will further investigate the relationship between autophagy and miR-205 to further confirm the mechanism underlying PR in EC. Future studies should also focus on other downstream actions of miR-205 in PR-EC. Nonetheless, because our observations are based on *in vitro* analyses, *in vivo* studies are also necessary.

## MATERIALS AND METHODS

### Materials

Human EC Ishikawa cells were obtained from the Chinese Academic of Science cell bank in Shanghai. Medroxyprogesterone acetate (MPA), dimethyl sulfoxide (DMSO) and methylthiazolyldiphenyl-tetrazolium bromide (MTT) were obtained from SIGMA (St. Louis, MO, USA). RPMI 1640 and fetal calf serum (FCS) were obtained from BRL Gibco (Carlsbad, CA, USA). Ethylenediaminetetraacetic acid (EDTA) and sodium carbonate (NaHCO_3_) were obtained from Amresco (OH, USA). Annexin-V/propidium iodine apoptosis detection kits were obtained from Bender Med Systems Inc. (Vienna, Austria). Penicillin/streptomycin, Dulbecco's Modified Eagle's Medium (DMEM), L-glutamine, streptomycin and trypsin were obtained from Invitrogen (Pontoise, France). Antibodies against LC3-II/I, PTEN, pAKT, pmTOR, beclin1 and GAPDH were purchased from Genscript Biotechnology (USA).

### Cell lines and culture conditions

A PR-EC sub-cell line (Ishikawa-PR cells) was obtained from parental Ishikawa cells via continuous exposure to increasing amounts of MPA dissolved in DMSO [32]. Ishikawa cells and Ishikawa-PR cells were cultured with RPMI 1640, supplemented with 10% FCS, 0.3 g/L L-glutamine, 100 U/mL penicillin, 100 l g/mL streptomycin, 0.85 g/L NaHCO_3_ and 101 g/mL insulin and incubated at 37°C in a humidified atmosphere supplemented with 5% CO_2_.

### Growth inhibition assay with MTT cell proliferation

Ishikawa-PR cells in the logarithmic growth phase were seeded into a 96-well plate at a concentration of 1 × 10^5^ cells/mL and treated with miR-205 inhibitor (WESTERN, Chongqing, China) or phosphate-buffered saline (PBS; control cells). The control and treated cells were harvested separately at 24, 48, and 72 h after treatment. Then, 10 μL 5 mg/mL MTT solution and 150 μL DMSO was added to each well, and absorbance was measured at 490 nm and 630 nm (Bio-Rad, Hercules, CA, USA). The assay was repeated at least three times.

### Cell cycle assay using propidium iodide with flow cytometry

To confirm the results of cell cycle analysis, we stained cells with propidium iodide and analyzed them by flow cytometry. Cells were cultured in 6-well plates to 60%–70% confluence; after 24, 48 or 72 h, the cells were transferred to 1.5 mL centrifuge tubes and centrifuged for 5 min at 2000 rpm. The cells were then fixed in an ice bath with 70% ethanol and incubated with 0.05 g/mL of RNase and 50 mg/mL propidium iodide. The cell samples were stained with 50 mg/mL propidium iodide (PI) at 37°C for 30 min after washing with PBS. The cycle cell was assessed at a wavelength of 488 nm using a Becton-Dickinson FACSort flow cytometer. The data collection and analysis were accomplished using Cell Quest and ModFit software.

### Apoptosis assessment using annexin V-FITC with flow cytometry

We stained cells using annexin V-FITC and evaluated them by flow cytometry according to the manufacturer's instructions (BD, San Diego, CA, USA). Cells at 60–80% confluence were seeded in a 6-well plate. After treatment with miR-205 inhibitor or PBS for 48 h, the collected cells were stained with 50 μL annexin-V-FITC and 10 μL PI and incubated at room temperature in the dark for 10 min. Flow cytometry was performed using excitation and emission wavelengths of 488 nm, 546 nm, 578 nm, and 647 nm. Each sample was examined to determine the percentage of cancer cells displaying annexin-V/PI (+/−) staining in the early apoptosis stage or annexin-V/PI (+/ +) staining in the late apoptosis or cell death stage.

### Quantitative real-time polymerase chain reaction (qRT-PCR)

Cells were lysed by replacing the medium with TRIzol reagent (Beyotime, China) and repeatedly pipetting the lysate until homogenous and completely lysed. The cell homogenate was incubated at room temperature for approximately 5 min until the nucleic acids and proteins were fully dissociated. Cold isopropanol was added to the homogenate, and the resulting precipitate was washed with 1 mL of 75% ethanol by gently shaking the tube to remove salt residue. The RNA precipitates were dissolved in an appropriate amount (20–50 μL) of RNase-free water. cDNA was synthesized from 5 μL total RNA using M-MLV Reverse Transcription Kit (Invitrogen, USA) with Bulge-loop miR-205 qRT-PCR Primer (synthesized by WESTERN, Chongqing, China). The sequence of hsa-miR-205 is UCCUUCAUUCCACCGGAGUCUG. The primers used to amplify miR-205 were 5′-TGCCGCCTGAACTTCACTCC-3′ (forward) and 5′-GAGCAGGCTGGAGAA-3′ (reverse). The primers used to amplify U6F were 5′-CTCGCTTCGGCAGCACATA-3′ (forward) and 5′-CGCTTCACGAATTTGCGTG-3′ (reverse). The amplification reactions were performed using SYBRGreen I (Western Biotechnology, China) according to the manufacturer's protocol. The melting curve and threshold cycle (Ct) data for each PCR product were determined.

The CT value represents the number of cycles required when for the fluorescent signal in the reaction tube to reach the set field value: ΔCt = Ct (target gene)-Ct (reference gene). The relative expression is 2 ^(−ΔCt)^, and the relative ratio is the relative copy number of the target gene/the relative copy number of the reference gene; therefore, the higher the relative expression or the relative ratio, the higher the mRNA expression of the target gene.

### Western blot analysis

After miR-205 inhibitor treatment, cells at 60–80% confluence were harvested and washed twice with PBS. The cell pellets were lysed with RIPA buffer and placed on ice for several minutes. Protein concentrations were detected using a BCA kit (Pierce Biological, Rockford, IL, USA) according to the manufacturer's instructions. The samples were separated by 12% sodium dodecyl sulfate-polyacrylamide electrophoresis and electro-blotted onto nitrocellulose membranes. The membranes were incubated with primary antibodies overnight at 4°C. Antisera against the following were used: LC3-II/I, PTEN, pAKT, pmTOR, or beclin1. After washing with blocking solution, the membranes were incubated with secondary antibodies for 1 h at 37°C, either a donkey anti-rabbit secondary antibody or a horseradish peroxidase-linked sheep anti-mouse antibody. Detected was performed using the ECL Reagent system (Amersham Pharmacia Biotech, Piscataway, NJ, USA).

### Statistical analyses

All data were collected from three independent experiments, and the results are expressed as the mean ± SE. Student's *t*-test was used to determine statistically significant differences between two groups. For comparisons of multiple groups, one-way ANOVA was used. The statistical analyses were performed using SPSS18.0 software. Differences between experimental and control groups were considered significant at a *p* value < 0.05.

## CONCLUSIONS

In this study, we found that inhibition of miR-205 induces cytotoxic effects in Ishikawa EC cells and Ishikawa-PR cells in a dose- and time-dependent manner. Suppression of cancer cell viability occurred through apoptosis alterations, cell cycle arrest and the autophagy pathway. Furthermore, miR-205 regulates cell growth by targeting AKT-mTOR signaling in PR-EC Ishikawa cells through activation of PTEN transcription and several autophagy-related proteins are expressed when miR-205 is inhibited. These data indicate that miR-205 may serve as a treatment for PR-EC, especially in a patient population hoping to preserve fertility. Finally, our observations provide new insight into the role of autophagy in anti-tumor therapy and progesterone resistance.

## ETHICAL APPROVAL

This article does not involve any studies with human participants performed by any of the authors.
